# Laparoscopic management of cystic duct cyst (Type VI): case report

**DOI:** 10.1093/jscr/rjag070

**Published:** 2026-02-14

**Authors:** José Luis Recalde Bravo, David Narváez Salas

**Affiliations:** Department of General Surgery, Hospital Vozandes Quito, Av. Juan José de Villalengua Oe2-37, 170521, Quito, Ecuador; Department of General Surgery, Hospital Vozandes Quito, Av. Juan José de Villalengua Oe2-37, 170521, Quito, Ecuador

**Keywords:** choledochal cyst, cystic duct cyst, acute pancreatitis, magnetic resonance cholangiopancreatography

## Abstract

Cystic duct cyst (CDC) is a rare biliary anomaly that may occur in isolation or in association with other choledochal cysts. While individual variants have been described, reports focusing on dual biliary pathology managed by a minimally invasive approach are limited. Surgical resection is recommended to prevent complications, including malignant transformation. We report the case of a 68-year-old woman with a remote history of cholecystectomy who presented with acute pancreatitis. Magnetic resonance cholangiopancreatography identified a CDC associated with dilatation of the common bile duct. Laparoscopic resection of the cysts and biliodigestive reconstruction were performed without complications. This case highlights the novelty of a dual biliary cystic pathology involving a Type VI CDC and a Type I choledochal cyst managed entirely by a laparoscopic approach, emphasizing the feasibility and safety of minimally invasive surgery in complex biliary anomalies.

## Introduction

Choledochal cysts (CCs) are congenital dilatations of the intra- and extrahepatic bile ducts. They are more frequently diagnosed in children, women, and Asian populations [1]. The increased availability and sophistication of imaging techniques have facilitated the identification of new variants of biliary duct cysts, including the cystic duct cyst (CDC) [2].

Clinical manifestations of CDCs are nonspecific and may include right upper quadrant mass, abdominal pain, jaundice, or acute pancreatitis (AP). Todani *et al*. classified CCs into five types based on the extent and morphology of biliary involvement [3]. The first description of a CDC dates to 1983, and its proposed addition as Type VI within the Todani classification was introduced in 1991 [4].

Over the past two decades, the number of CC cases reported in adults has increased, leading to rising recognition of this entity. Although Type VI cysts are not formally included in Todani’s original classification, they are described as dilatations of the cystic duct [5]. Approximately 50 cases have been documented in PUBMED, of which around 60% involve isolated CDCs, while the remainder feature concomitant common bile duct (CBD) involvement.

We present a case of Type VI CDC associated with a fusiform Type IC CC, successfully managed via laparoscopic resection and biliodigestive reconstruction.

## Case report

A 68-year-old woman with a medical history of hypothyroidism, paroxysmal tachycardia treated with two ablations, and an episode of AP in 2023 was evaluated. Her surgical history included laparoscopic cholecystectomy 20 years earlier and laparoscopic appendectomy 16 years earlier.

**Figure 1:**
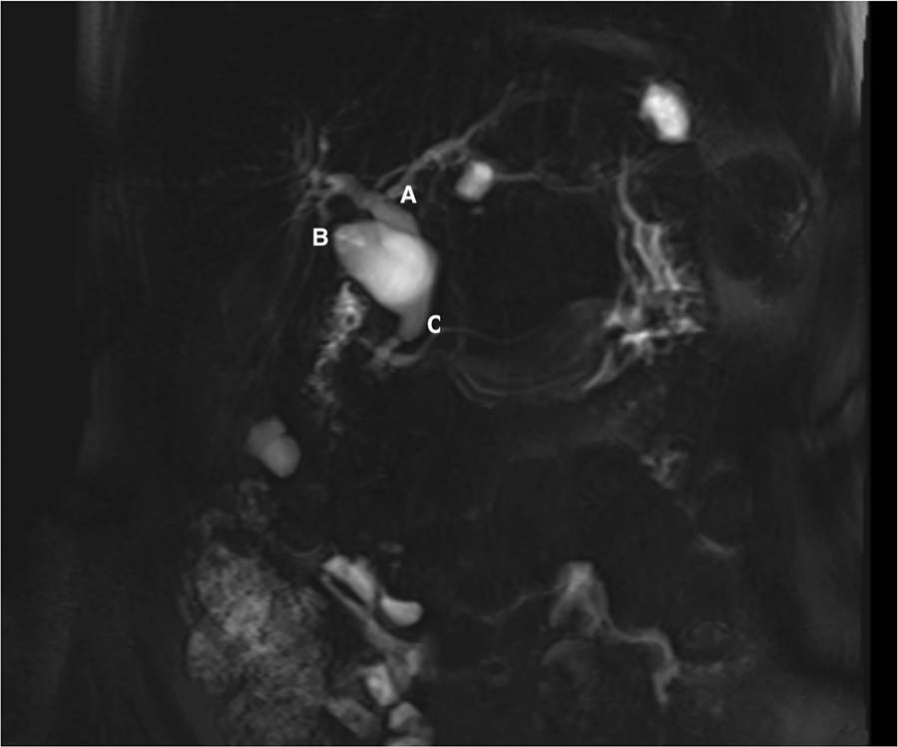
MRCP showing the common hepatic duct (A), cystic duct dilatation of 23 mm (B), and common bile duct of 14 mm (C).

Following her pancreatitis episode, MRCP was performed to identify a possible etiology. Imaging revealed cyst dilatation of the cystic duct and concomitant CBD dilatation (Fig. 1). Physical examination showed no jaundice or palpable masses. Laboratory tests were within normal limits.

**Figure 2:**
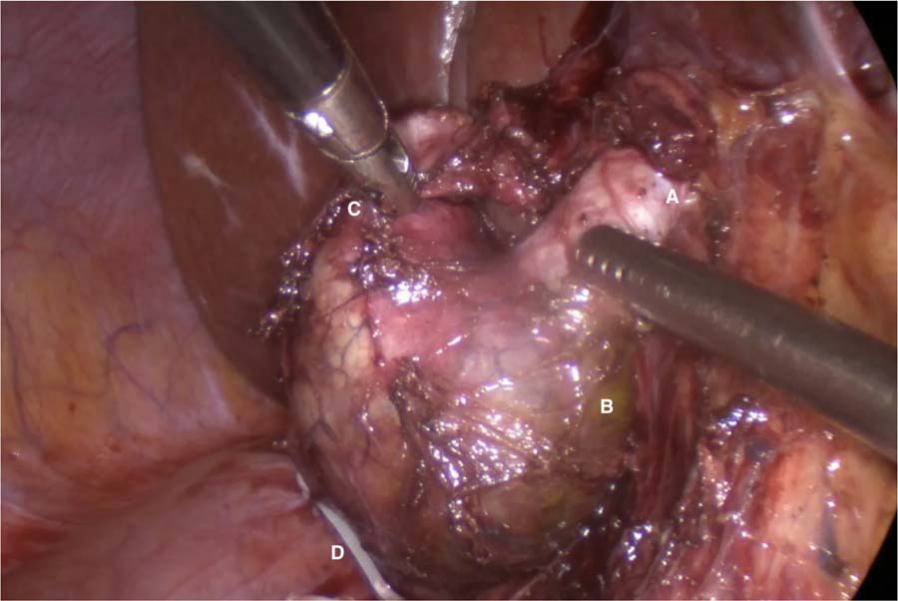
Common hepatic duct (A), fusiform dilatation of the common bile duct (B), cystic duct cyst (C), polymeric clip on the distal portion of the common bile duct once the distal dissection and resection at this level has been completed (D).

**Figure 3:**
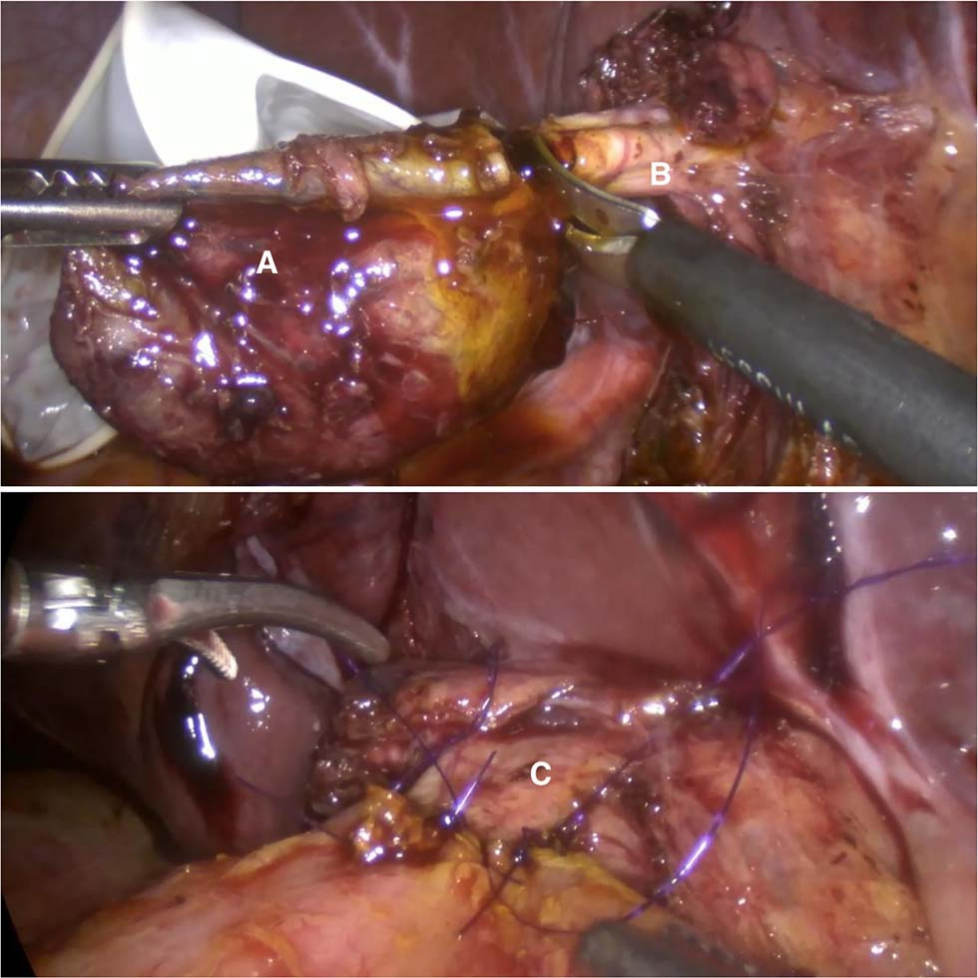
Complete excision of the cyst (A) at the level of the common hepatic duct (B); biliodigestive derivation conformation (C).

Given the imaging findings and previous AP, laparoscopic surgical management was indicated. Intraoperatively, a fusiform Type IC CC and a Type VI CDC were confirmed (Fig. 2). Complete laparoscopic excision of both cysts was performed, followed by biliodigestive reconstruction (Fig. 3).

## Discussion

CDC (Type VI) is a distinct biliary anomaly that poses diagnostic and therapeutic challenges, particularly when associated with other CCs, and its diagnosis and management continue to be subjects of debate. Similar to other CCs, CDCs are more commonly reported in Asian populations, particularly in India [5]. Kilambi *et al*. reported that 38% of CDC cases occur in association with other biliary abnormalities, most commonly Type I and Type IV CCs [6], as seen in our patient.

The most widely accepted pathophysiological mechanism for CCs involves an anomalous pancreaticobiliary junction, predisposing to reflux of pancreatic enzymes into the biliary tree, resulting in inflammation, increased intraluminal pressure, and ductal dilatation [3]. Baytok and Ecer proposed that CDC formation may be associated with congenital weakness of the cystic duct wall and absence of myenteric ganglion cells, producing a diverticulum-like outpouching [7].

Symptoms typically include abdominal pain, jaundice, cholangitis, or an abdominal mass. Ronnekleiv-Kelly *et al*. reported abdominal pain in 61%, jaundice in 16%, AP in 18.5%, and asymptomatic presentation in 16% of cases [1]. In the present case, AP prompted further diagnostic evaluation.

Imaging—particularly MRCP—is essential to define the anatomy and guide surgical planning. Despite its advantages, MRCP may fail to diagnose CDC in up to one-third of cases, likely due to rarity and interpreter unfamiliarity [5].

There is no consensus on the optimal management of CDC. When isolated, treatment typically consists of cholecystectomy with complete excision of the cystic duct. In the presence of CBD involvement, complete excision of both the CDC and CC followed by biliodigestive reconstruction is recommended to reduce the risk of malignancy and prevent pancreaticobiliary reflux [6].

The risk of malignant transformation within the biliary tree is a well-recognized long-term complication of CC. According to De Kleine *et al*., the overall incidence of malignancy is ~11%, with the highest prevalence observed in Type I and Type IV CC. This risk can be significantly reduced to around 0.3% following complete surgical excision. However, due to the limited number of reported CDC, precise estimates of malignancy risk remain poorly defined. At present, there is no consensus regarding an optimal or standardized long-term surveillance strategy [8].

## Conclusions

This case further demonstrates that a minimally invasive approach is feasible even in complex biliary anatomy and supports laparoscopic excision as a safe and effective strategy for dual biliary pathology.
